# HbA_1c_, diabetes and cognitive decline: the English Longitudinal Study of Ageing

**DOI:** 10.1007/s00125-017-4541-7

**Published:** 2018-01-25

**Authors:** Fanfan Zheng, Li Yan, Zhenchun Yang, Baoliang Zhong, Wuxiang Xie

**Affiliations:** 1 Brainnetome Center, Institute of Automation, Chinese Academy of Sciences, Beijing, People’s Republic of China; 2 Institute of Cognitive Neuroscience, University College London, London, UK; 3 Department of Epidemiology and Biostatistics, School of Public Health, Imperial College London, London, UK; 4 Affiliated Wuhan Mental Health Center, Tongji Medical College of Huazhong University of Science and Technology, Wuhan, People’s Republic of China; 5 Peking University Clinical Research Institute, Peking University Health Science Center, No. 38 Xueyuan Road, Haidian District, 100191 Beijing, People’s Republic of China

**Keywords:** Cognitive decline, Diabetes, HbA_1c_, Trajectory

## Abstract

**Aims/hypothesis:**

The aim of the study was to evaluate longitudinal associations between HbA_1c_ levels, diabetes status and subsequent cognitive decline over a 10 year follow-up period.

**Methods:**

Data from wave 2 (2004–2005) to wave 7 (2014–2015) of the English Longitudinal Study of Ageing (ELSA) were analysed. Cognitive function was assessed at baseline (wave 2) and reassessed every 2 years at waves 3–7. Linear mixed models were used to evaluate longitudinal associations.

**Results:**

The study comprised 5189 participants (55.1% women, mean age 65.6 ± 9.4 years) with baseline HbA_1c_ levels ranging from 15.9 to 126.3 mmol/mol (3.6–13.7%). The mean follow-up duration was 8.1 ± 2.8 years and the mean number of cognitive assessments was 4.9 ± 1.5. A 1 mmol/mol increment in HbA_1c_ was significantly associated with an increased rate of decline in global cognitive *z* scores (−0.0009 SD/year, 95% CI −0.0014, −0.0003), memory *z* scores (−0.0005 SD/year, 95% CI −0.0009, −0.0001) and executive function *z* scores (−0.0008 SD/year, 95% CI −0.0013, −0.0004) after adjustment for baseline age, sex, total cholesterol, HDL-cholesterol, triacylglycerol, high-sensitivity C-reactive protein, BMI, education, marital status, depressive symptoms, current smoking, alcohol consumption, hypertension, CHD, stroke, chronic lung disease and cancer. Compared with participants with normoglycaemia, the multivariable-adjusted rate of global cognitive decline associated with prediabetes and diabetes was increased by −0.012 SD/year (95% CI −0.022, −0.002) and −0.031 SD/year (95% CI −0.046, −0.015), respectively (*p* for trend <0.001). Similarly, memory, executive function and orientation *z* scores showed an increased rate of cognitive decline with diabetes.

**Conclusions/interpretation:**

Significant longitudinal associations between HbA_1c_ levels, diabetes status and long-term cognitive decline were observed in this study. Future studies are required to determine the effects of maintaining optimal glucose control on the rate of cognitive decline in people with diabetes.

**Electronic supplementary material:**

The online version of this article (10.1007/s00125-017-4541-7) contains peer-reviewed but unedited supplementary material, which is available to authorised users.


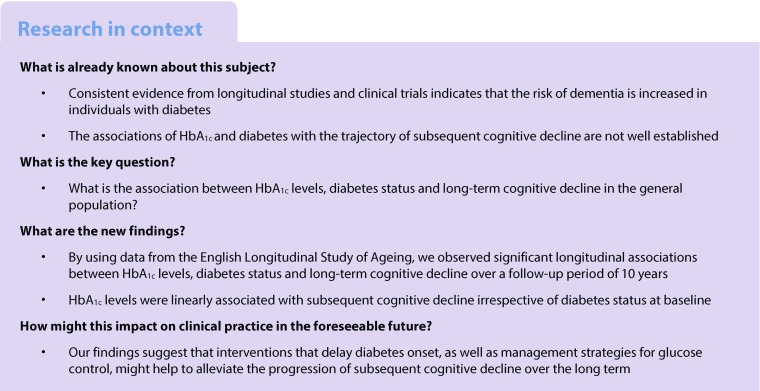


## Introduction

Of the psychiatric conditions strongly associated with poor quality of later life, dementia is one of the most prevalent and thus represents a serious public health burden, given the substantial increase in ageing populations around the world [1–3]. Diabetes, a metabolic disease characterised by hyperglycaemia that can lead to long-term dysfunction, has rapidly increased in prevalence over the past several decades [4, 5]. The association between diabetes and dementia has been well documented [6–8]; however, the association between diabetes and cognitive decline is less well studied. Cognitive decline occurs over a long period prior to dementia, and the trajectory of cognitive decline, which consists of several assessments of cognitive function, is important for evaluating and monitoring the progression of cognitive deterioration. Identification of risk factors for cognitive decline could help screen individuals who may benefit from early intervention.

HbA_1c_ is central to the management of glucose control in individuals with diagnosed diabetes and has recently been recommended for use in diagnosing diabetes as well as identifying people at risk of developing diabetes [9]. Compared with individual fasting or post-load blood glucose measurements, HbA_1c_ has better reliability between tests [10] because it reflects average circulating glucose levels over the preceding 2–3 months, making it a stronger predictor of subsequent diabetes [11]. However, there is little research prospectively investigating the association of hyperglycaemia, based on HbA_1c_ levels, with long-term cognitive decline in individuals with and without diabetes [12, 13]. Moreover, the studies conducted on this topic to date use cognitive trajectories that consist of only three cognitive assessments [12, 13].

The English Longitudinal Study of Ageing (ELSA) presents an opportunity to investigate the association between HbA_1c_ levels and the subsequent trajectory of cognitive decline based on a greater number of cognitive assessments. Therefore, the objectives of the present study were: (1) to determine whether prediabetes (defined by an HbA_1c_ level in the range 38.8–46.4 mmol/mol [5.7–6.4%] [14]) and/or diabetes are associated with accelerated cognitive decline in an elderly population with normal cognition at baseline; and (2) to examine whether people with diabetes with better glycaemic control, as measured by HbA_1c_ levels, show a similar or decreased rate of subsequent cognitive decline.

## Methods

### Study population

**Fig. 1:**
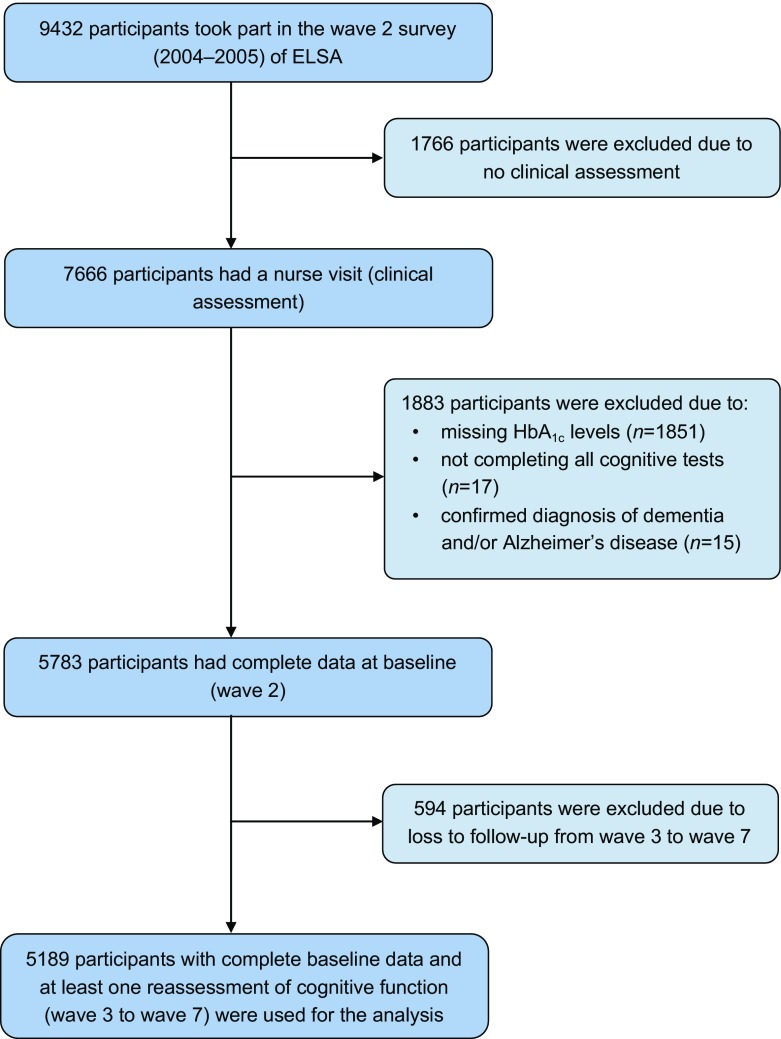
Flow chart of participant selection for the present study population

This study used data from wave 2 (2004–2005) to wave 7 (2014–2015) of the ELSA, a prospective and nationally representative cohort of men and women living in England aged 50 years and over [15]. A detailed description of the goals, design and methods of the ELSA has been published elsewhere [16]. A flow chart of participant selection for the present study population based on inclusion and exclusion criteria is shown in Fig. 1. A total of 9432 individuals attended the wave 2 survey of the ELSA. Of these, 1766 were excluded from the present study because they did not have a nurse visit (clinical assessment). A further 1883 individuals were excluded for the following reasons: they had missing HbA_1c_ results (*n* = 1851), they did not complete all of the cognitive tests (*n* = 17) or they had a confirmed diagnosis of dementia and/or Alzheimer’s disease at baseline (*n* = 15). An additional 594 individuals were excluded from the main analysis (but were included in a sensitivity analysis) because they were lost to follow-up from waves 3 to 7. The remaining 5189 participants (2329 men and 2860 women) with complete baseline data and at least one reassessment of cognitive function (waves 3–7) were included in the analyses reported here.

The ELSA was approved by the London Multicentre Research Ethics Committee (MREC/01/2/91). Informed consent was obtained from all participants.

### Cognitive assessments

Participants underwent a memory assessment through immediate and delayed recall of ten unrelated words. Both immediate and delayed recall scores ranged from 0 to 10, with higher scores indicating better memory performance. Immediate and delayed recall tests have been shown to have good construct validity and consistency [17]. A composite memory score was created by summing the scores of the two individual memory tests. Executive function was assessed by a verbal fluency task in which participants were required to orally name as many animals as they could in 60 s. The task is well documented to be both reliable and valid, and has previously been used as an indicator of executive function for the ELSA population [18]. The score for this task was the total number of words produced, excluding repeat words and non-animal words. Orientation was assessed by asking four questions regarding the date, i.e. day of month, month, year and day of week, and scoring one point for each correct answer. Generally, higher scores indicate better cognitive function.

To enable comparison across cognitive tests, *z* scores standardised to wave 2 were generated for individual tests by subtracting the mean score at wave 2 from the participant’s test score at each wave and dividing by the SD of the wave 2 scores. A composite global cognitive *z* score was calculated for each participant by averaging the *z* scores of the three tests and re-standardising to wave 2 using the mean and SD of the global cognitive *z* score at wave 2. A *z* score of 1 would therefore describe cognitive performance that is 1 SD above the mean score at wave 2. For all cognitive tests, we used standardised values in the regression analysis to allow for comparisons of regression coefficients across cognitive tests.

### Measurement of HbA_1c_

In wave 2, blood samples were collected and sent to the Biochemistry Department at the Royal Victoria Infirmary, Newcastle, UK for laboratory analysis [19]. Total HbA_1c_ was measured by the Haematology Department at the Royal Victoria Infirmary using a Tosoh G7 analyser (Tosoh, Tokyo, Japan) [19]. The analytical methods used for HbA_1c_ measurement in the UK are required to be traceable to the work carried out in the Diabetes Control and Complications Trial (DCCT), part of the National Glycohemoglobin Standardization Program in the USA. The Secondary Reference Laboratory at the University of Minnesota was the main analytical laboratory for the DCCT work.

### Definition of diabetes and prediabetes

Diabetes was defined as an HbA_1c_ level ≥47.5 mmol/mol (6.5%), a self-reported physician diagnosis of diabetes or current use of glucose-lowering therapy. Among participants without diabetes, we defined prediabetes as an HbA_1c_ level in the range 38.8–46.4 mmol/mol (5.7–6.4%), according to the 2014 American Diabetes Association guidelines [4]. In participants with diabetes, HbA_1c_ levels were further categorised using a standard clinical cut-off value of 53.0 mmol/mol (7.0%) to test the effect of glucose management on subsequent cognitive decline [4].

### Covariates

Covariates shown by previous studies to be associated with both HbA_1c_ levels and cognitive function were selected for our analyses. These covariates included age, sex, total cholesterol, HDL-cholesterol, triacylglycerol, circulating high-sensitivity C-reactive protein (CRP), BMI, education, marital status, depressive symptoms, current smoking, alcohol consumption, hypertension, CHD, stroke, chronic lung disease and cancer. Details of covariates are available in the ESM Methods.

### Statistical analysis

The results are presented as percentages for categorical variables and means ± SDs for normally distributed continuous variables. The results for high-sensitivity CRP and triacylglycerol are presented as medians with interquartile ranges because their distribution was highly skewed. The cross-sectional associations between HbA_1c_ levels and cognitive scores at baseline were tested using multiple linear regression models, and linear mixed models were used to evaluate longitudinal associations. We also conducted longitudinal analyses to calculate the mean difference in the rate of change in cognitive scores (SD/year) and compared categories of baseline diabetes status using non-diabetic participants with normal HbA_1c_ levels (<38.8 mmol/mol [5.7%]) as the reference group. Linear mixed models can incorporate all available follow-up data, account for the fact that repeated measures in the same participant are correlated with each other, and handle missing data. In the two models that we ran, both the intercept and the slope were fitted as random effects to account for inter-individual differences at baseline and different rates of change in cognitive function over the follow-up period. The first model included HbA_1c_ levels (or diabetes status), time (years since baseline), time × HbA_1c_ interaction, age (years) and sex (male or female). The time × HbA_1c_ interaction term indicated differential change by each one unit increment in HbA_1c_ from baseline to the end of the study. The second model additionally adjusted for baseline total cholesterol (mmol/l), HDL-cholesterol (mmol/l), triacylglycerol (mmol/l), high-sensitivity CRP (nmol/l), BMI (kg/m^2^), education (below level 3 National Vocational Qualification [NVQ3]/General Certificate of Education [GCE] A level, or above or equal to NVQ3/GCE A level), marital status (currently living alone or not), depressive symptoms (yes or no), current smoking (yes or no), one or more alcoholic drinks once or more per week (yes or no), hypertension (yes or no), CHD (yes or no), stroke (yes or no), chronic lung disease (yes or no) and cancer (yes or no).

We used a multiple imputation, chained-equations method to replace missing data for cognitive assessments during follow-up (waves 3–7) and used all available data from 5783 participants in the sensitivity analyses. Variables used to impute the missing values of cognitive scores included participants’ baseline information (age, sex, education, marital status, BMI, current smoking, alcohol consumption, diabetes and stroke) and baseline cognitive scores. For each longitudinal analysis, we created 20 imputed data sets and combined the results using the MIANALYZE procedure of SAS version 9.4 (SAS Institute, Cary, NC, USA). To detect differences in the rate of change in cognitive *z* scores between individuals with diabetes and those completely free of diabetes, we conducted another sensitivity analysis that excluded 261 participants with incident diabetes during follow-up.

Statistical analyses were performed using SAS software, version 9.4 (SAS Institute). All analyses were two-sided; an alpha value of 0.05 was considered the threshold for statistical significance.

## Results

### Baseline characteristics and sample size

The mean age of the 5189 participants was 65.6 ± 9.4 years; 55.1% of participants were women. Within the sample, 1190 participants (22.9%) were classified as having prediabetes and 446 (8.6%) were classified as having diabetes. The mean HbA_1c_ level across all participants was 37.7 ± 7.9 mmol/mol (5.57 ± 0.79%) and ranged from 15.9 to 126.3 mmol/mol (3.6 to 13.7%). The distribution of baseline covariates and cognitive scores by diabetes status is shown in Table 1.

**Table 1:** Characteristics of the study participants at baseline (wave 2), according to baseline diabetes status

Characteristic	Baseline characteristic by diabetes status			*p* for trend^a^
	Normal (*n*=3553)	Prediabetes (*n*=1190)	Diabetes (*n*=446)	
Age, years	64.8 ± 9.1	67.4 ± 9.9	67.6 ± 8.7	<0.001
Women, %	1995 (56.1)	665 (55.9)	200 (44.8)	0.001
HbA_1c_, mmol/mol	34.4 ± 2.7	40.7 ± 1.9	55.6 ± 15.0	<0.001
HbA_1c_, %	5.26 ± 0.43	5.87 ± 0.18	7.23 ± 1.39	<0.001
Total cholesterol, mmol/l	6.03 ± 1.12	5.99 ± 1.28	4.99 ± 1.13	<0.001
HDL-cholesterol, mmol/l	1.58 ± 0.39	1.48 ± 0.36	1.29 ± 0.33	<0.001
Triacylglycerol, mmol/l	1.4 (1.0–2.0)	1.7 (1.2–2.4)	1.9 (1.4–2.8)	<0.001
High-sensitivity CRP, nmol/l	16.2 (7.6–34.3)	24.8 (12.4–47.6)	22.9 (12.4–46.7)	<0.001
BMI, kg/m^2^	27.2 ± 4.4	28.7 ± 4.8	30.2 ± 4.9	<0.001
Systolic BP, mmHg	135.0 ± 18.4	137.6 ± 18.7	138.7 ± 18.4	<0.001
Diastolic BP, mmHg	76.1 ± 10.5	75.8 ± 11.5	73.7 ± 11.5	<0.001
Education NVQ3/GCE A level or above, %	1283 (36.1)	342 (28.7)	129 (28.9)	<0.001
Living alone, %	1093 (30.8)	421 (35.4)	154 (34.5)	0.006
Depressive symptoms, %	431 (12.1)	184 (15.5)	81 (18.2)	<0.001
Current smoking, %	426 (12.0)	226 (19.0)	68 (15.2)	<0.001
Alcoholic drink once or more per week, %	2232 (62.8)	608 (51.1)	187 (41.9)	<0.001
Hypertension, %	1554 (43.7)	583 (49.0)	256 (57.4)	<0.001
CHD, %	144 (4.1)	113 (9.5)	70 (15.7)	<0.001
Stroke, %	46 (1.3)	35 (2.9)	19 (4.3)	<0.001
Chronic lung disease, %	152 (4.3)	70 (5.9)	29 (6.5)	0.006
Cancer, %	194 (5.5)	49 (4.1)	25 (5.6)	0.406
Memory score	10.5 ± 3.4	10.0 ± 3.5	9.4 ± 3.3	<0.001
Executive function score	20.8 ± 6.3	20.1 ± 6.1	19.4 ± 6.7	<0.001
Orientation score	3.78 ± 0.49	3.78 ± 0.53	3.76 ± 0.49	0.435

Values are mean ± SD, median (interquartile range) or *n* (%)

^a^Calculated using a linear regression analysis or χ^2^ test for trend

From waves 2 to 7, the cohort size was 5189, 4969, 4378, 4045, 3732 and 3276, respectively. The mean follow-up duration was 8.1 ± 2.8 years and the mean number of cognitive assessments was 4.9 ± 1.5.

### Baseline HbA_1c_ levels, diabetes status and cognitive scores (cross-sectional analyses)

Linear regression analyses found that baseline HbA_1c_ levels were significantly associated with global cognitive, memory and executive function *z* scores after adjustment for age and sex, but these associations lost significance after further adjustment for total cholesterol, HDL-cholesterol, triacylglycerol, high-sensitivity CRP, BMI, education, marital status, depressive symptoms, current smoking, alcohol consumption, hypertension, CHD, stroke, chronic lung disease and cancer (ESM Table 1). In both models, orientation scores were not linearly associated with HbA_1c_ levels. As shown in ESM Fig. 1, none of the cognitive scores were significantly associated with baseline diabetes status after multivariable adjustment.

### Baseline HbA_1c_ levels, diabetes status and cognitive decline (longitudinal analyses)

Table 2 shows the longitudinal associations between HbA_1c_ levels and rate of change in cognitive scores. After multivariable adjustment, a 1 mmol/mol increment in HbA_1c_ was significantly associated with an increased rate of decline in global cognitive *z* scores (−0.0009 SD/year, 95% CI −0.0014, −0.0003), memory *z* scores (−0.0005 SD/year, 95% CI −0.0009, −0.0001) and executive function *z* scores (−0.0008 SD/year, 95% CI −0.0013, −0.0004), but not orientation *z* scores (−0.0004 SD/year, 95% CI −0.0011, 0.0002).

**Table 2:** Longitudinal analysis of the association between baseline HbA_1c_ levels (per 1 mmol/mol increment) and rate of change in cognitive *z* scores (SD/year), using linear mixed models

*z* Score	Model 1^a^		Model 2^b^	
	β (95% CI)	*p* value	β (95% CI)	*p* value
Global cognitive *z* score	−0.0009 (−0.0014, −0.0003)	0.002	−0.0009 (−0.0014, −0.0003)	0.002
Memory *z* score	−0.0005 (−0.0009, −0.0001)	0.019	−0.0005 (−0.0009, −0.0001)	0.025
Executive function *z* score	−0.0009 (−0.0013, −0.0004)	<0.001	−0.0008 (−0.0013, −0.0004)	<0.001
Orientation *z* score	−0.0004 (−0.0011, 0.0002)	0.167	−0.0004 (−0.0011, 0.0002)	0.186

^a^Model 1: adjusted for baseline age and sex

^b^Model 2: further adjusted for baseline total cholesterol, HDL-cholesterol, triacylglycerol, high-sensitivity CRP, BMI, education, marital status, depressive symptoms, current smoking, alcohol consumption, hypertension, CHD, stroke, chronic lung disease and cancer

**Fig. 2:**
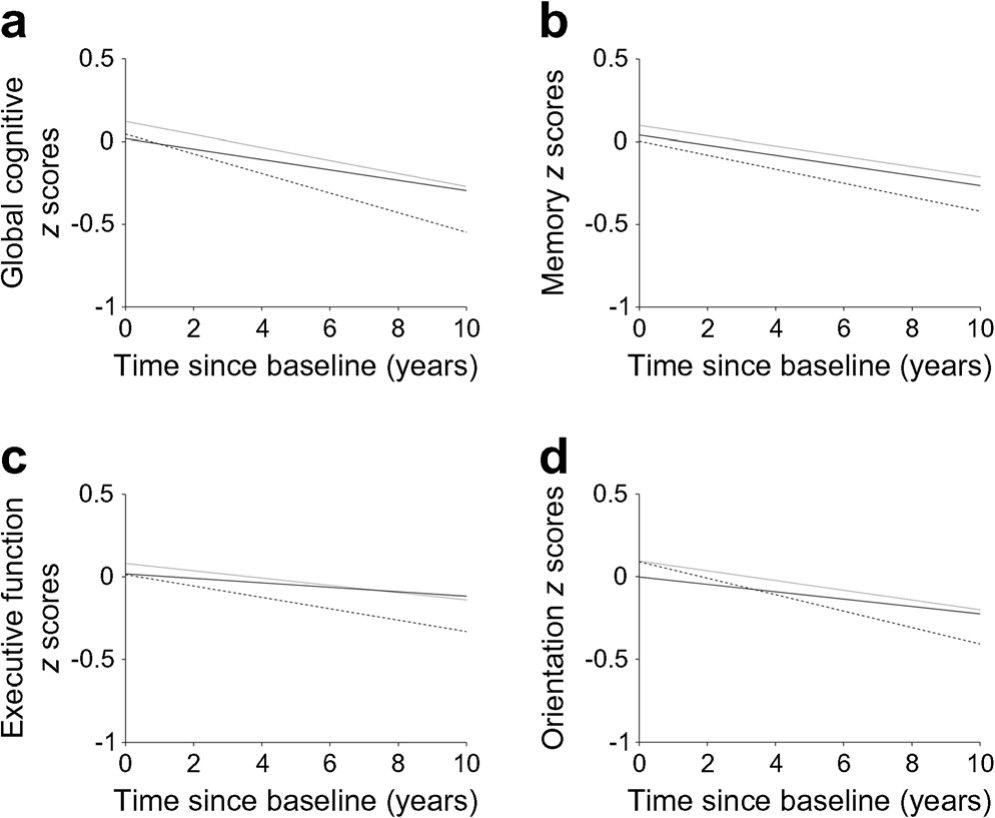
Trajectories of cognitive *z* scores by baseline diabetes status (solid lines, normal; dotted lines, prediabetes; dashed lines, diabetes), adjusted for baseline age, sex, total cholesterol, HDL-cholesterol, triacylglycerol, high-sensitivity CRP, BMI, education, marital status, depressive symptoms, current smoking, alcohol consumption, hypertension, CHD, stroke, chronic lung disease and cancer

As shown in Fig. 2, the cognitive scores of participants with diabetes decreased at a greater rate than those of non-diabetic participants with normal HbA_1c_ levels (<38.8 mmol/mol [5.7%]). The multivariable-adjusted rates of global cognitive decline associated with prediabetes and diabetes were increased by −0.012 SD/year (95% CI −0.022, −0.002) and −0.031 SD/year (95% CI −0.046, −0.015), respectively (*p* for trend <0.001; Table 3), compared with the normal group. Similarly, memory, executive function and orientation *z* scores also declined at a greater rate in participants with diabetes (Table 3). Compared with the normal group, the multivariable-adjusted rate of memory decline associated with diabetes was increased by −0.015 SD/year (95% CI −0.026, −0.003), and the rates of executive function and orientation decline were increased by −0.022 SD/year (95% CI −0.034, −0.009) and −0.023 SD/year (95% CI −0.041, −0.006), respectively (Table 3).

**Table 3:** Longitudinal analysis of mean difference in rate of change in cognitive *z* scores (SD/year) comparing categories of baseline diabetes status, using linear mixed models

	Mean difference (95% CI) in rate of change by baseline diabetes status			*p* for trend
	Normal (*n*=3553)	Prediabetes (*n*=1190)	Diabetes (*n*=446)	
Global cognitive *z* score				
Model 1^a^	0.000 (ref.)	−0.013 (−0.022, −0.003)	−0.031 (−0.046, −0.015)	<0.001
Model 2^b^	0.000 (ref.)	−0.012 (−0.022, −0.002)	−0.031 (−0.046, −0.015)	<0.001
Memory *z* score				
Model 1^a^	0.000 (ref.)	−0.002 (−0.009, 0.005)	−0.015 (−0.026, −0.004)	0.026
Model 2^b^	0.000 (ref.)	−0.002 (−0.009, 0.005)	−0.015 (−0.026, −0.003)	0.030
Executive function *z* score				
Model 1^a^	0.000 (ref.)	−0.008 (−0.016, 0.000)	−0.022 (−0.034, −0.009)	<0.001
Model 2^b^	0.000 (ref.)	−0.008 (−0.016, 0.000)	−0.022 (−0.034, −0.009)	<0.001
Orientation *z* score				
Model 1^a^	0.000 (ref.)	−0.011 (−0.023, −0.000)	−0.023 (−0.040, −0.005)	0.003
Model 2^b^	0.000 (ref.)	−0.011 (−0.022, 0.000)	−0.023 (−0.041, −0.006)	0.003

^a^Model 1: adjusted for baseline age and sex

^b^Model 2: further adjusted for baseline total cholesterol, HDL-cholesterol, triacylglycerol, high-sensitivity CRP, BMI, education, marital status, depressive symptoms, current smoking, alcohol consumption, hypertension, CHD, stroke, chronic lung disease and cancer

We further categorised diabetic participants into two groups using a standard clinical cut-off value for HbA_1c_ (53.0 mmol/mol [7.0%]). Among participants with diabetes and an HbA_1c_ level <53.0 mmol/mol (7.0%), the mean HbA_1c_ level was 45.7 ± 4.7 mmol/mol (6.33 ± 0.43%) and the median level was 46.5 mmol/mol (6.4%). Among those with diabetes and an HbA_1c_ level ≥53.0 mmol/mol (7.0%), the mean HbA_1c_ level was 67.2 ± 14.6 mmol/mol (8.30 ± 1.34%) and the median level was 61.8 mmol/mol (7.8%). As shown in ESM Fig. 2, global cognitive *z* scores of participants with diabetes and an HbA_1c_ level ≥53.0 mmol/mol (7.0%) declined fastest during the follow-up. The overall decreased trend of cognitive decline remained significant among the four groups (*p* for trend <0.001). However, there was no significant difference in the rate of global cognitive decline between the two diabetic groups (*p* = 0.453; ESM Fig. 2). Compared with participants with diabetes and an HbA_1c_ level <53.0 mmol/mol (7.0%), the multivariable-adjusted rate of global cognitive decline associated with diabetic participants with an HbA_1c_ level ≥53.0 mmol/mol (7.0%) showed a non-significant increase of −0.007 SD/year (95% CI −0.023, 0.036). In addition, we divided 446 participants with diabetes into two groups according to whether they were taking glucose-lowering therapies at baseline. The results showed that glucose-lowering therapies were not significantly associated with future cognitive decline (ESM Table 2).

### Non-response analyses

From the completed ELSA cohort, 3649 individuals (38.7%) were excluded from this study because of incomplete baseline data or a confirmed diagnosis of dementia and/or Alzheimer’s disease. This group of excluded participants had: a higher percentage of women; were living alone; had depressive symptoms; were smokers; had self-reported diabetes, CHD or stroke; a lower percentage of high education and alcohol consumption; and poorer cognitive function (ESM Table 3). An additional 594 individuals (6.3%), who were excluded because of loss to follow-up, also had higher levels of the major risk factors and poorer cognitive function (ESM Table 4).

### Sensitivity analyses

Longitudinal analysis results using imputed data (*n* = 5783) were similar to those from the main analyses (ESM Tables 5 and 6). Thus, the impact of missing data on our main findings was likely to be small. Likewise, another sensitivity analysis showed similar results to those of the main analysis when 261 participants who became diabetic during follow-up were excluded (ESM Tables 7 and 8).

## Discussion

In this community-based population, we observed a significant trend for cognitive decline over a 10 year period among individuals aged ≥50 years with normoglycaemia, prediabetes or diabetes at baseline. Additionally, HbA_1c_ levels were linearly associated with subsequent cognitive decline in memory and executive function (but not orientation) irrespective of diabetes status at baseline.

To the best of our knowledge, this is the first prospective study of the association between diabetes (assessed using HbA_1c_ levels) and cognitive decline that analyses data from more than three cognitive assessments over time. From these data, we were able to generate a reliable and accurate trajectory of cognitive decline with which to investigate this association. Our results are compatible with prior studies exploring this association using clinical categories of HbA_1c_ levels [12, 13]. Two cohort studies, both conducted in middle-aged populations, reported significantly faster cognitive decline in participants with diabetes than in those with normoglycaemia, although the tests used to measure cognitive function were different from those used in our study [12, 13]. Regarding the relationship between prediabetes and cognitive decline, results are inconsistent between studies. In agreement with the present study, Tuligenga et al reported that cognitive decline was not significantly faster in people with prediabetes than in those with normoglycaemia [13]. Conversely, Rawlings et al reported that cognitive decline was significantly faster among people with prediabetes than among those with normal HbA_1c_ levels [12]. Given the similar trends of cognitive decline among participants with normoglycaemia, prediabetes and diabetes in all three of the aforementioned studies, it is possible that sample size is responsible for the controversial results. In particular, the significant result for cognitive decline in people with prediabetes vs those with normoglycaemia was obtained from a cohort of 2365 individuals with prediabetes [12], whereas the non-significant results were obtained from cohorts of only 648 and 1190 individuals with prediabetes in the study by Tuligenga et al [13] and the present study, respectively. Further studies with larger sample sizes are required to validate the association between prediabetes and cognitive decline.

According to recommendations of the American Diabetes Association, maintaining an HbA_1c_ level of less than 53.0 mmol/mol (7.0%) could help prevent diabetes-related microvascular complications [9]. Therefore, using HbA_1c_ levels as a marker of glucose management, we divided our diabetic participants into two groups to examine the effect of glucose management on subsequent cognitive decline. The results revealed that while both groups showed a significant trend towards cognitive decline, there was no significant difference between the two groups. This result agrees with that of a previous study reporting a greater but not statistically significant decline in diabetic participants with an HbA_1c_ level of at least 53.0 mmol/mol (7.0%) [12]. This finding might be attributable to diabetes treatment-related adverse events such as severe hypoglycaemia [20], which would result in fluctuations in blood glucose levels. Although the underlying mechanisms remain to be elucidated, it is suggested that both hyperglycaemia and hypoglycaemia play important roles in diabetes-related cognitive decline [21, 22]. Studies of cellular mechanisms suggest that, compared with sustained hyperglycaemia, glycaemic fluctuations may have a greater adverse effect on endothelial function and induce more oxidative stress, potentially leading to greater cognitive decline [23, 24]. However, findings from randomised clinical trials are conflicting. No effect on cognitive decline was observed following an intervention to reduce HbA_1c_ levels in the Action to Control Cardiovascular Risk in Diabetes (ACCORD) Memory in Diabetes Study (ACCORD-MIND) [25], while slower cognitive decline was observed following an intervention at an HbA_1c_ level of 53.0 mmol/mol (7.0%) or less in the Informatics in Diabetes Education and Telemedicine Study (IDEATel) [26]. Additionally, no benefits for cognitive function were reported by the Anglo–Danish–Dutch Study of Intensive Treatment in People with Screen-Detected Diabetes in Primary Care–Netherlands (ADDITION-Netherlands) in their comparison of the effectiveness of intensive multifactorial treatment (including keeping HbA_1c_ levels lower than 53.0 mmol/mol [7.0%]) with routine care [27]. However, all the studies mentioned above emphasise the importance of early intervention to prevent or delay diabetes onset. Modest cognitive function decrements are already present during the early stage of diabetes [28], and the effects of tight glycaemic control on those with established diabetes are complicated and yet to be determined. In addition, HbA_1c_ levels were recently recommended to be used in clinical practice in the UK, to assess diabetes and serve as a biomarker for blood glucose management according to the guidelines of the National Institute for Health and Care Excellence [29]. This is consistent with our opinion that HbA_1c_ levels are important for the prevention and management of diabetes. Given that HbA_1c_ levels were not used in clinical practice during the conduct of the present study, we also investigated whether fasting glucose levels and HbA_1c_ levels produced different diabetes diagnoses. As shown in ESM Table 9, HbA_1c_ levels identified more diabetic participants than did fasting glucose levels; the mismatch rate was approximately 1.4% in total. Therefore, the effect of mismatch between different diagnosis standards on our results is likely to be minimal.

Notably, the observed linear correlation of HbA_1c_ levels with global cognitive decline was primarily driven by impairments in the domains of memory and executive function, which were assessed by immediate and delayed word recall tests and a verbal fluency task, respectively. This may suggest that cognitive decline related to high circulating glucose levels could be specific to dysfunction of certain brain regions or subcortical pathways involved in memory and executive function. Another possible explanation is that the orientation test, with only four questions and a score ranging from 0 to 4, was relatively insensitive to the small increments of cognitive decline induced by high glucose levels. Nevertheless, it is still worth mentioning that, although the observed associations were statistically significant, the effect sizes were quite small.

While the precise mechanisms underlying the association of diabetes with cognitive decline remain unclear, several potential mechanisms have been proposed. Diabetes has been implicated to be related to subsequent cognitive impairment through both direct mechanisms (e.g. by inducing amyloid accumulation) and indirect mechanisms (e.g. by increasing microvascular disease of the central nervous system—believed to play a very important role in vascular dementia) [8]. In addition, individuals with diabetes are at increased risk of comorbidities such as depression, obesity, hypertension and hyperlipidaemia, all of which could affect cognitive performance [30]. However, only small attenuations of associations were noted after adjustment for such factors, indicating that diabetes is an independent and strong risk factor for cognitive decline. A critical aspect of successful ageing is maintaining cognitive function and ensuring a high quality of life. It has been shown that even a modest decrease in cognitive function could result in substantially greater cognitive decline over several years [31]. As there is currently no cure for dementia, early intervention on modifiable risk factors, such as diabetes, may offer an important way to prevent cognitive decline. Indeed, it has been established that intervention for treating and addressing modifiable risk factors for dementia could prevent up to a quarter of dementia cases [30].

A major strength of the present study is that it is one of the largest general population-based studies exploring the relationship between HbA_1c_ levels and cognition over a long-term 10 year follow-up period. Another strength is that we obtained repeated measures of cognitive function over the follow-up period, providing a robust assessment of cognitive deterioration and enabling us to capture the cumulative burden and chronicity of estimates of long-term trajectories of cognitive decline. Nevertheless, the present findings should be considered in the context of some potential limitations. First, the extent to which we can infer a causal relationship between HbA_1c_ levels and cognitive decline is limited because of the observational study design. It has been argued that even longitudinal designs cannot completely assuage this criticism. However, our findings show that high HbA_1c_ levels were not independently related to poorer cognitive function at baseline, but to a greater longitudinal cognitive decline, thus implying that poor cognition is a corollary of high HbA_1c_ levels, but not vice versa. Second, our study lacked a clinical dementia diagnosis during follow-up; therefore, we cannot analyse the temporal relationship between HbA_1c_ levels, diabetes and incident dementia. Third, although we adjusted for many potential confounding factors, there may be residual confounding factors such as genetic susceptibility, including the *APOE* genotype. Genetic data are not available for the ELSA and so we cannot adjust for the *APOE* genotype; however, previous studies indicated that there is no interaction with *APOE* status and diabetes on cognitive decline [7, 8, 32]. Fourth, only 55.0% of participants who completed the wave 2 survey were eligible for this study, which might have led to selection bias. Non-response analyses show that the study sample was healthier than the original ELSA population, which may affect the internal validity of estimates and limit generalisation to the English population. Using only the responders might have diluted the association between baseline diabetes and future cognitive decline, because dropouts and non-responders had a higher percentage of self-reported diabetes and might have had an even faster cognitive decline compared with responders. Thus, a stronger association could be expected if there had been full participation. Finally, cognitive function was assessed using isolated tasks; a more elaborate neuropsychological assessment may result in different associations.

In conclusion, our study provides evidence to support the association of diabetes with subsequent cognitive decline. Moreover, our findings show a linear correlation between circulating HbA_1c_ levels and cognitive decline, regardless of diabetic status. Future studies are required to determine the long-term effects of maintaining optimal glucose control on cognitive decline in people with diabetes.

## Electronic supplementary material

ESM (PDF 591 kb)

## Data Availability

The data that support the findings of this study are available from the corresponding author upon reasonable request.

## Abbreviations

CRP: C-reactive protein
ELSA: English Longitudinal Study of Ageing
GCE: General Certificate of Education
NVQ3: Level 3 National Vocational Qualification
