# Supplementary material for: HbA1c, diabetes and cognitive decline: the English Longitudinal Study of Ageing
Source: Diabetologia. 2018 Jan 25;61(4):839–48. doi: 10.1007/s00125-017-4541-7 (PMC6448974; doi:10.1007/s00125-017-4541-7)
Supplement: Supplementary file 1 — (PDF 591 kb) [file 125_2017_4541_MOESM1_ESM.pdf]

Electronic supplementary material

Fanfan Zheng, Li Yan, Zhenchun Yang, Baoliang Zhong, Wuxiang Xie. Glycated haemoglobin, diabetes and cognitive decline: the English Longitudinal Study of Ageing. *Diabetologia.*

Methods

Table 1 Linear associations between baseline glycated haemoglobin levels (per one unit increment, %) and cognitive *Z* scores: cross-sectional analyses using multiple linear regressions

Table 2 Mean difference in rate of change in cognitive Z scores (SD/year) between diabetes participants with and without anti-diabetic therapies at baseline: longitudinal analyses using linear mixed models

Table 3 Comparison of baseline characteristics between participants included (n=5189), and excluded due to incomplete baseline data or confirmed diagnosis of dementia and/or Alzheimer's disease (n=3649)

Table 4 Comparison of baseline characteristics between participants included (n=5189) and excluded due to loss to follow-up (n=594)

Table 5 Association between baseline glycated haemoglobin levels (per one unit increment, %) and rate of change in cognitive Z scores (SD/year): longitudinal analyses using linear mixed models, with multiple imputation for missing data for cognitive tests at wave 3 to wave 7, n=5783

Table 6 Mean difference in rate of change in cognitive Z scores (SD/year) comparing categories of baseline diabetes status: longitudinal analyses using linear mixed models, with multiple imputation for missing data for cognitive tests at wave 3 to wave 7, n=5783

Table 7 Association between baseline glycated haemoglobin levels (per one unit increment, %) and rate of change in cognitive Z scores (SD/year): longitudinal analyses using linear mixed models, a sensitivity analysis excluding 261 participants with incident diabetes during follow-up, n=4928

Table 8 Mean difference in rate of change in cognitive Z scores (SD/year) comparing categories of baseline diabetes status: longitudinal analyses using linear mixed models, a sensitivity analysis excluding 261 participants with incident diabetes during follow-up, n=4928

Table 9 Categories of diabetes based on different diagnosis standards

Fig. 1 Baseline cognitive *Z* scores and 95% confidence intervals by diabetes status

Fig. 2 (A) The trajectories of global cognitive *Z* scores by baseline diabetes status and HbA1c levels; (B) difference in global cognitive *Z* scores decline by baseline diabetes status and HbA_{1c} levels compared with decline in participants with normoglycaemia

Methods

Covariates

Total cholesterol (Cholesterol Oxidase assay method), high-density lipoprotein cholesterol (direct method), and triacylglycerol (enzymatic method) levels were measured using the Olympus 640 analyser calibrated to the center for disease control guidelines. Circulating high-sensitivity C-reactive protein (CRP) was assessed using the N latex CRP mono immunoassay on the Dade Behring Nephelometer II Analyser and conducted in line with the quality control guidelines specified in the Health Survey of England technical report [1]. Blood pressure was measured by the nurse on the right arm of each participant while they were in a sitting position, using the Omron HEM-907 [1]. Five minutes elapsed before the first reading was taken. The mean value of three consecutive blood pressure readings was used in our analyses. Hypertension was considered as a systolic blood pressure of ≥140 mm Hg and/or a diastolic blood pressure of ≥90 mm Hg, or if the participant was currently using anti-hypertensive drugs. Education level was classified as no qualification, level 1 national vocational qualification (NVQ) or certificate of secondary education, NVQ2 or O-level, NVQ3 or A-level, higher qualification but below degree, and degree level or higher or NVQ4/5. Marital status was classified as single (never married), married, remarried, legally separated, divorced, and widowed. We defined cohabitation status as currently living alone or not. Participants were split into two groups: non-smokers (never smoked or ex-smokers) and smokers (current smokers). Alcohol intake was calculated from participant-reported drinking frequency over the previous year (weekly drinking versus occasional or never). Standing height was measured with a portable stadiometer, with participants standing in the center of the base plate looking straight ahead, and weight was measured using a portable electronic scales [1]. Body mass index was

calculated with the following formula: weight (kg) / height^2 (m^2). Depressive symptoms were measured with the eight-item version of the Center for Epidemiologic Studies Depression Scale, a widely used self-report measure of depressive symptoms, used to identify people at risk of depression in population-based studies. As in previous studies, we used a score of ≥4 to define cases of elevated depressive symptoms [2]. Measures of chronic disease included lifetime self-reported physician diagnoses of coronary heart disease (angina and heart attack), stroke, chronic lung disease, and cancer.

References

1. Graig R, Deverill C, Pickering K (2006) Quality control of blood saliva and urine analytes. In: Spronston k,mindell j, editors. Health survey for england 2004, methodology and documentation, vol. 2. London: The information centre
2. Hamer M, Batty GD, Kivimaki M (2012) Risk of future depression in people who are obese but metabolically healthy: The English Longitudinal Study of Ageing. Mol Psychiatry 17:940-945

Table 1 Linear associations between baseline glycated haemoglobin levels (per one unit increment, %) and cognitive *Z* scores: cross-sectional analyses using multiple linear regressions

	Model 1[a]		Model 2[b]	
Baseline cognitive *Z* scores	*β* (95% CI)	*P* value	*β* (95% CI)	*P* value
Global cognitive *Z* scores	−0.054 (−0.089, −0.020)	0.002	−0.015 (−0.050, 0.020)	0.396
Memory *Z* scores	−0.071 (−0.105, −0.036)	<0.001	−0.032 (−0.067, 0.004)	0.080
Executive function *Z* scores	−0.052 (−0.089, −0.016)	0.005	−0.015 (−0.052, 0.022)	0.423
Orientation *Z* scores	0.007 (−0.030, 0.045)	0.702	0.014 (−0.025, 0.054)	0.478

[a]Model 1: adjusted for baseline age and sex.

[b]Model 2: further adjusted for baseline total cholesterol, high-density lipoprotein cholesterol, triacylglycerol, high-sensitivity C-reactive protein, body mass index, education, marital status, depression symptoms, current smoking, alcohol consumption, hypertension, coronary heart disease, stroke, chronic lung disease, and cancer.

Table 2 Mean difference in rate of change in cognitive Z scores (SD/year) between diabetes participants with and without anti-diabetic therapies at baseline: longitudinal analyses using linear mixed models

	Mean difference (95% CI) in rate of change in cognitive Z scores (SD/year)		
	Diabetes without treatments (n=189)	Diabetes with treatments (n=257)	*P* for difference
Global cognitive *Z* scores			
Model 1[a]	0.000 (ref)	0.017 (−0.012, 0.046)	0.250
Model 2[b]	0.000 (ref)	0.018 (−0.011, 0.047)	0.230
Memory *Z* scores			
Model 1[a]	0.000 (ref)	0.003 (−0.020, 0.026)	0.786
Model 2[b]	0.000 (ref)	0.003 (−0.020, 0.026)	0.798
Executive function *Z* scores			
Model 1[a]	0.000 (ref)	−0.006 (−0.029, 0.018)	0.632
Model 2[b]	0.000 (ref)	−0.005 (−0.029, 0.018)	0.667
Orientation *Z* scores			
Model 1[a]	0.000 (ref)	0.028 (−0.005, 0.061)	0.096
Model 2[b]	0.000 (ref)	0.028 (−0.005, 0.060)	0.101

[a]Model 1: adjusted for baseline age and sex.

[b]Model 2: further adjusted for baseline total cholesterol, high-density lipoprotein cholesterol, triacylglycerol, high-sensitivity C-reactive protein, body mass index, education, marital status, depression symptoms, current smoking, alcohol consumption, hypertension, coronary heart disease, stroke, chronic lung disease, and cancer.

Table 3 Comparison of baseline characteristics between participants included (n=5189), and excluded due to incomplete baseline data or confirmed diagnosis of dementia and/or Alzheimer's disease (n=3649)

Characteristic	Included (n=5189)	Excluded (n=3649)	*P* for difference*
Age (years)	65.6±9.4	65.4±12.3	0.354
Women (%)	2860 (55.1)	2148 (58.9)	<0.001
Education ≥ NVQ3/GCE A level (%)	1754 (33.8)	1034 (28.3)	<0.001
Living alone (%)	1668 (32.1)	1294 (35.5)	0.001
Depressive symptoms (%)	696 (13.4)	633 (17.4)	<0.001
Current smoking (%)	720 (13.9)	659 (18.1)	<0.001
Alcoholic drink ≥ once per week (%)	3027 (58.3)	1667 (45.7)	<0.001
Self-reported diagnosis of diabetes	358 (6.9)	346 (9.5)	<0.001
Coronary heart disease (%)	327 (6.3)	304 (8.3)	<0.001
Stroke (%)	100 (1.9)	170 (4.7)	<0.001
Chronic lung disease (%)	251 (4.8)	200 (5.5)	0.176
Cancer (%)	268 (5.2)	200 (5.5)	0.513
Memory scores	10.3±3.4	9.6±3.8	<0.001
Executive function scores	20.5±6.3	19.1±7.1	<0.001
Orientation scores	3.78±0.49	3.67±0.76	<0.001

The results are presented as mean ± SD or n (%).

*The differences between participants included and excluded were tested using the *t*-test or chi-square test.

Table 4 Comparison of baseline characteristics between participants included (n=5189) and excluded due to loss to follow-up (n=594)

Characteristic	Included (n=5189)	Loss to follow-up (n=594)	*P* for difference*
Age (years)	65.6±9.4	69.5±11.3	<0.001
Women (%)	2860 (55.1)	298 (50.2)	0.022
Glycated haemoglobin (mmol/mol)	37.4±8.6	37.8±9.1	0.335
Glycated haemoglobin (%)	5.57±0.79	5.60±0.83	0.335
Total cholesterol (mmol/l)	5.93±1.20	5.77±1.23	0.001
HDL cholesterol (mmol/l)	1.53±0.39	1.48±0.37	0.001
Triacylglycerol (mmol/l)	1.5 (1.1–2.2)	1.5 (1.1–2.2)	0.839
High-sensitivity CRP (nmol/l)	18.1 (8.6–39.0)	22.9 (10.5–54.3)	<0.001
Body mass index (kg/m^2)	27.8±4.6	27.6±4.7	0.478
Systolic blood pressure (mm Hg)	135.9±18.5	138.5±20.6	0.003
Diastolic blood pressure (mm Hg)	75.8±10.8	75.1±12.2	0.159
Education ≥ NVQ3/GCE A level (%)	1754 (33.8)	119 (20.0)	<0.001
Living alone (%)	1668 (32.1)	200 (33.7)	0.450
Depressive symptoms (%)	696 (13.4)	96 (16.2)	0.065
Current smoking (%)	720 (13.9)	96 (16.2)	0.129
Alcoholic drink ≥ once per week (%)	3027 (58.3)	288 (48.5)	<0.001
Hypertension (%)	2393 (46.1)	305 (51.4)	0.015
Diabetes (%)	446 (8.6)	64 (10.8)	0.076
Coronary heart disease (%)	327 (6.3)	49 (8.3)	0.068
Stroke (%)	100 (1.9)	24 (4.0)	<0.001
Chronic lung disease (%)	251 (4.8)	38 (6.4)	0.098
Cancer (%)	268 (5.2)	40 (6.7)	0.106
Memory scores	10.3±3.4	8.8±3.7	<0.001
Executive function scores	20.5±6.3	18.0±6.5	<0.001
Orientation scores	3.78±0.49	3.66±0.70	<0.001

The results are presented as mean ± SD, median (quartile 1–quartile 3), or n (%).

*The differences between participants included and excluded were tested using the *t*-test, Wilcoxon rank test or chi-square test.

Table 5 Association between baseline glycated haemoglobin levels (per one unit increment, %) and rate of change in cognitive *Z* scores (SD/year): longitudinal analyses using linear mixed models, with multiple imputation for missing data for cognitive tests at wave 3 to wave 7, n=5783

	Model 1[a]		Model 2[b]	
	β (95% CI)	*P* value	*β* (95% CI)	*P* value
Global cognitive *Z* scores	−0.009 (−0.015, −0.003)	0.002	−0.009 (−0.015, −0.003)	0.003
Memory *Z* scores	−0.005 (−0.009, −0.001)	0.024	−0.005 (−0.009, −0.001)	0.036
Executive function *Z* scores	−0.008 (−0.013, −0.004)	<0.001	−0.008 (−0.013, −0.003)	0.001
Orientation *Z* scores	−0.004 (−0.011, 0.002)	0.209	−0.004 (−0.011, 0.003)	0.241

[a]Model 1: adjusted for baseline age and sex.

[b]Model 2: further adjusted for total cholesterol, high-density lipoprotein cholesterol, triacylglycerol, high-sensitivity C-reactive protein, body mass index, education, marital status, depression symptoms, current smoking, alcohol consumption, hypertension, coronary heart disease, stroke, chronic lung disease, and cancer.

Table 6 Mean difference in rate of change in cognitive *Z* scores (SD/year) comparing categories of baseline diabetes status: longitudinal analyses using linear mixed models, with multiple imputation for missing data for cognitive tests at wave 3 to wave 7, n=5783

	Mean difference (95% CI) in rate of change by baseline diabetes status			
	Normal (n=3553)	Prediabetes (n=1190)	Diabetes (n=446)	*P* for trend
Global cognitive *Z* scores				
Model 1[a]	0.000 (ref)	−0.011 (−0.021, −0.002)	−0.031 (−0.046, −0.016)	<0.001
Model 2[b]	0.000 (ref)	−0.011 (−0.021, −0.001)	−0.031 (−0.046, −0.016)	<0.001
Memory *Z* scores				
Model 1[a]	0.000 (ref)	−0.003 (−0.010, 0.004)	−0.016 (−0.027, −0.005)	0.012
Model 2[b]	0.000 (ref)	−0.003 (−0.010, 0.005)	−0.015 (−0.027, −0.004)	0.016
Executive function *Z* scores				
Model 1[a]	0.000 (ref)	−0.009 (−0.017, −0.002)	−0.024 (−0.036, −0.011)	<0.001
Model 2[b]	0.000 (ref)	−0.009 (−0.017, −0.001)	−0.023 (−0.035, −0.011)	<0.001
Orientation *Z* scores				
Model 1[a]	0.000 (ref)	−0.009 (−0.020, 0.002)	−0.022 (−0.040, −0.005)	0.004
Model 2[b]	0.000 (ref)	−0.009 (−0.020, 0.003)	−0.024 (−0.041, −0.006)	0.005

[a]Model 1: adjusted for baseline age and sex.

[b]Model 2: further adjusted for baseline total cholesterol, high-density lipoprotein cholesterol, triacylglycerol, high-sensitivity C-reactive protein, body mass index, education, marital status, depression symptoms, current smoking, alcohol consumption, hypertension, coronary heart disease, stroke, chronic lung disease, and cancer.

Table 7 Association between baseline glycated haemoglobin levels (per one unit increment, %) and rate of change in cognitive Z scores (SD/year): longitudinal analyses using linear mixed models, a sensitivity analysis excluding 261 participants with incident diabetes during follow-up, n=4928

	Model 1[a]		Model 2[b]	
	β (95% CI)	*P* value	*β* (95% CI)	*P* value
Global cognitive *Z* scores	−0.010 (−0.016, −0.004)	0.002	−0.010 (−0.016, −0.004)	0.002
Memory *Z* scores	−0.005 (−0.010, −0.001)	0.021	−0.005 (−0.009, −0.001)	0.026
Executive function *Z* scores	−0.009 (−0.014, −0.005)	<0.001	−0.009 (−0.014, −0.004)	<0.001
Orientation *Z* scores	−0.004 (−0.011, 0.003)	0.234	−0.004 (−0.011, 0.003)	0.256

[a]Model 1: adjusted for baseline age and sex.

[b]Model 2: further adjusted for baseline total cholesterol, high-density lipoprotein cholesterol, triacylglycerol, high-sensitivity C-reactive protein, body mass index, education, marital status, depression symptoms, current smoking, alcohol consumption, hypertension, coronary heart disease, stroke, chronic lung disease, and cancer.

Table 8 Mean difference in rate of change in cognitive Z scores (SD/year) comparing categories of baseline diabetes status: longitudinal analyses using linear mixed models, a sensitivity analysis excluding 261 participants with incident diabetes during follow-up, n=4928

	Mean difference (95% CI) in rate of change by baseline diabetes status			
	Normal (n=3553)	Prediabetes (n=1190)	Diabetes (n=446)	*P* for trend
Global cognitive *Z* scores				
Model 1[a]	0.000 (ref)	−0.014 (−0.025, −0.004)	−0.031 (−0.046, −0.015)	<0.001
Model 2[b]	0.000 (ref)	−0.014 (−0.024, −0.003)	−0.031 (−0.046, −0.015)	<0.001
Memory *Z* scores				
Model 1[a]	0.000 (ref)	−0.001 (−0.009, 0.006)	−0.015 (−0.027, −0.004)	0.027
Model 2[b]	0.000 (ref)	−0.001 (−0.009, 0.006)	−0.015 (−0.026, −0.004)	0.030
Executive function *Z* scores				
Model 1[a]	0.000 (ref)	−0.009 (−0.017, −0.000)	−0.022 (−0.034, −0.009)	<0.001
Model 2[b]	0.000 (ref)	−0.009 (−0.017, −0.000)	−0.021 (−0.034, −0.009)	<0.001
Orientation *Z* scores				
Model 1[a]	0.000 (ref)	−0.011 (−0.023, 0.002)	−0.022 (−0.040, −0.004)	0.004
Model 2[b]	0.000 (ref)	−0.010 (−0.022, 0.002)	−0.023 (−0.041, −0.005)	0.005

[a]Model 1: adjusted for baseline age and sex.

[b]Model 2: further adjusted for baseline total cholesterol, high-density lipoprotein cholesterol, triacylglycerol, high-sensitivity C-reactive protein, body mass index, education, marital status, depression symptoms, current smoking, alcohol consumption, hypertension, coronary heart disease, stroke, chronic lung disease, and cancer.

Table 9 Categories of diabetes based on different diagnosis standards.

Diabetes_2[b]	Diabetes[a]		
	No	Yes	Total
No	2965	28	2993
Yes	18	375	393
Total	2983	403	3386[c]

[a]Diabetes was defined as an HbA1c level ≥47.5 mmol/mol (6.5%), a self-reported physician diagnosis of diabetes, or current use of anti-diabetic therapy.
[b]Diabetes_2 was defined as a fasting blood glucose level ≥7.0 mmol/l, a self-reported physician diagnosis of diabetes, or current use of anti-diabetic therapy.
[c]Only 3386 participants have provided data of fasting glucose levels.

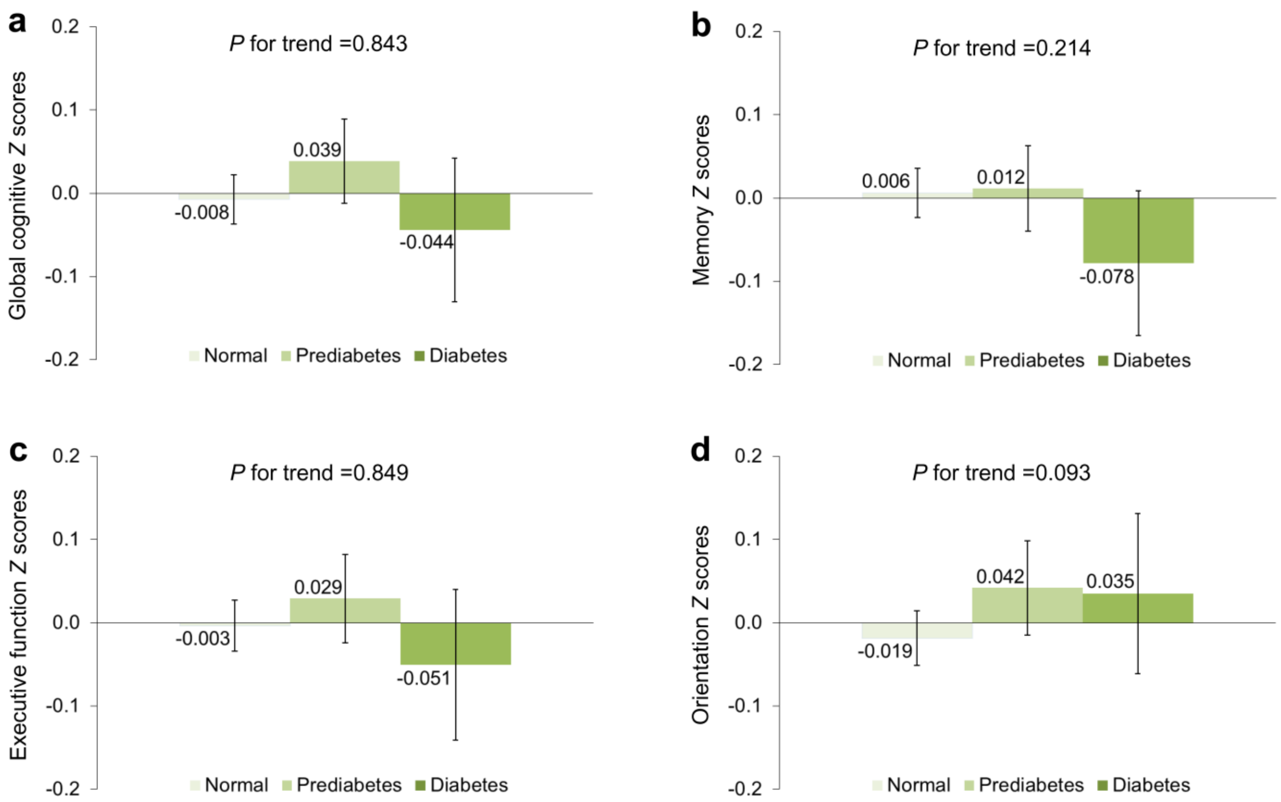

Fig. 1 Baseline cognitive *Z* scores and 95% confidence intervals by diabetes status. Cross-sectional analyses using analyses of covariance, adjusted for baseline age, sex, total cholesterol, high-density lipoprotein cholesterol, triacylglycerol, high-sensitivity CRP, body mass index, education, marital status, depression symptoms, current smoking, alcohol consumption, hypertension, coronary heart disease, stroke, chronic lung disease, and cancer.

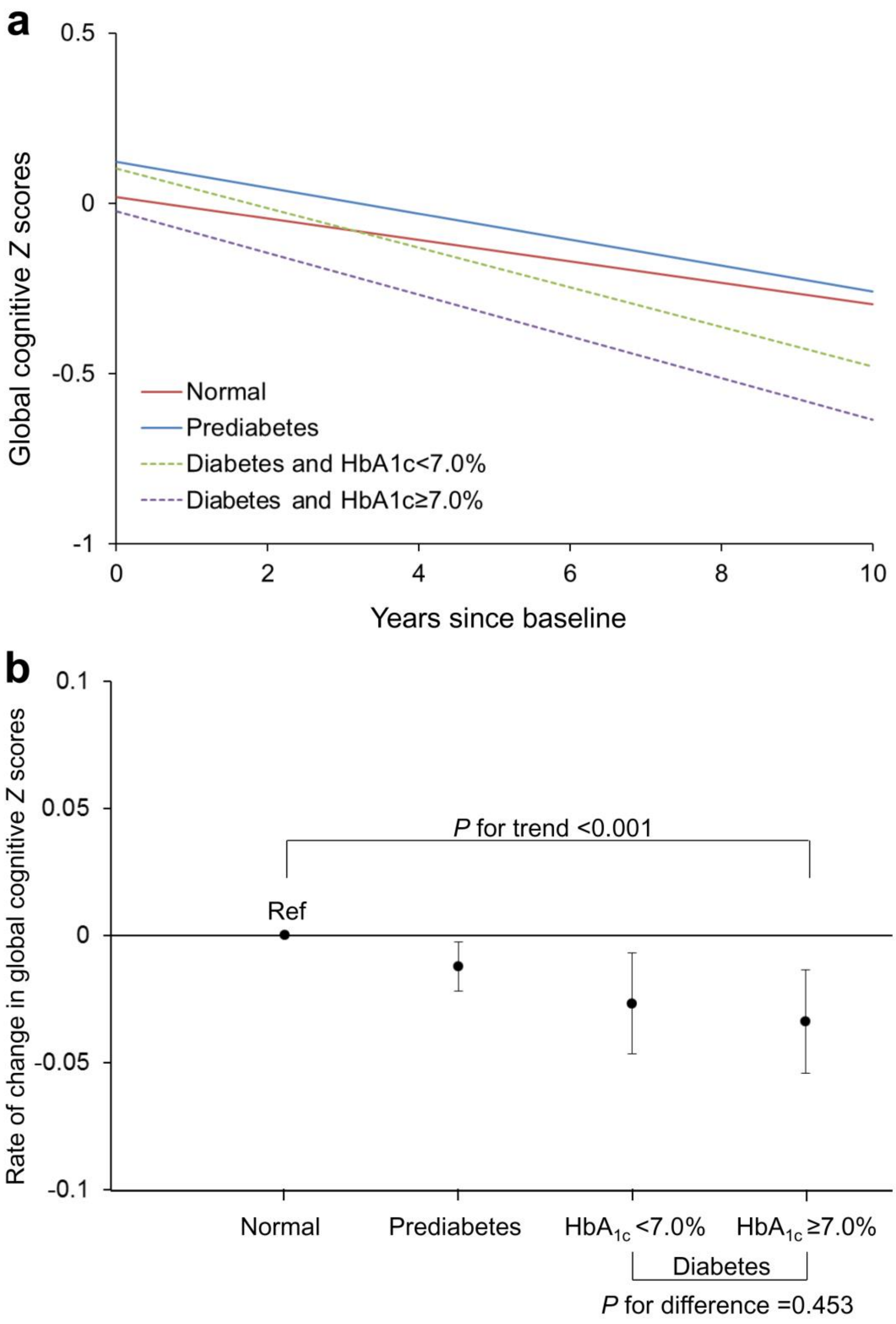

Fig. 2 (A) The trajectories of global cognitive *Z* scores by baseline diabetes status and HbA_{1c} levels; (B) difference in global cognitive *Z* scores decline (SD/year) by baseline diabetes status and HbA_{1c} levels compared with decline in participants with normoglycemia, adjusting for baseline age, sex, total cholesterol, high-density lipoprotein cholesterol, triacylglycerol, high-sensitivity CRP, body mass index, education, marital status, depression symptoms, current smoking, alcohol consumption, hypertension, coronary heart disease, stroke, chronic lung disease, and cancer.
